# Rubber without Sulphur

**Published:** 1868-02

**Authors:** 


*Rubber without Sulphur.*—Our attention has recently been called to a new preparation of dental rubber which is said to harden by the escape of the fumes of the bromine and iodine with which it is mixed. No sulphur is used in its preparation; it is not hardened by steam, but by dry heat, and its proprietors assert that it is far purer than the vulcanite and has neither taste nor smell, and does not change color during the hardening process.

Not having obtained as yet a sample of this new preparation, we are not able to judge of its merits. It is known by the name of Dried or Iodized Rubber.

